# (*E*)-*N*′-(4-Nitro­benzyl­idene)-4-(8-quinol­yloxy)butano­hydrazide

**DOI:** 10.1107/S1600536810020039

**Published:** 2010-06-05

**Authors:** Guo-Lun XiaHou, Ye-Chun Ding, Xiao-Na Fan

**Affiliations:** a Key Lab of Natural Medicine Research and Development in Jiangxi, Gannan Medical University, Ganzhou, Jiangxi 341000, People’s Republic of China

## Abstract

In the title compound, C_20_H_18_N_4_O_4_, conformation along the bond sequence linking the benzene and quinoline rings, which have a mean inter­planar dihedral angle of 2.7 (5)°, is *trans–*(+)*gauche–trans–trans–*(−)*gauche–trans–trans*. In the crystal structure, a pair of inter­molecular N—H⋯O hydrogen bonds links the mol­ecules into centrosymmetric cyclic *R*_2_^2^(8) dimers, which are aggregated *via* π–π inter­actions into parallel sheets [quinoline–benzene ring centroid separation = 3.6173 (16)–3.6511 (16) Å]. The sheets are further connected through weak C—H⋯O inter­actions, giving a supra­molecular two-dimensional network.

## Related literature

For general background to Schiff bases in coordination chemistry, see: Calligaris & Randaccio (1987 ▶). For related structures, see: Zheng, Li *et al.* (2008 ▶); Zheng, Qiu *et al.* (2006 ▶); Zheng, Wu, Lu *et al.* (2006 ▶); Zheng (2006 ▶); Zheng, Wu, Li *et al.* (2006 ▶, 2007 ▶); Xie *et al.* (2008 ▶); Chen & Li (2009 ▶); Zhang *et al.* (2009 ▶). For bond-length data, see: Allen *et al.* (1987 ▶). For hydrogen-bond motifs, see: Bernstein *et al.* (1995 ▶). 
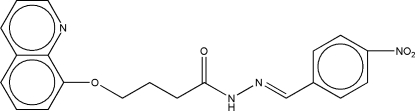


## Experimental

### 

#### Crystal data

C_20_H_18_N_4_O_4_
*M* *_r_* = 378.38
Monoclinic, 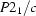

*a* = 9.836 (3) Å
*b* = 10.633 (3) Å
*c* = 17.566 (5) Å
β = 92.365 (7)°
*V* = 1835.6 (9) Å^3^
*Z* = 4
Mo *K*α radiation
μ = 0.10 mm^−1^
*T* = 296 K
0.22 × 0.17 × 0.15 mm

#### Data collection

Bruker SMART CCD area-detector diffractometer
Absorption correction: multi-scan (*SADABS*; Sheldrick, 1996 ▶) *T*_min_ = 0.979, *T*_max_ = 0.986
9957 measured reflections
3256 independent reflections
2345 reflections with *I* > 2σ(*I*)
*R*_int_ = 0.036

#### Refinement

*R*[*F* ^2^ > 2σ(*F* ^2^)] = 0.044
*wR*(*F* ^2^) = 0.146
*S* = 1.07
3256 reflections
253 parameters
H-atom parameters constrained
Δρ_max_ = 0.22 e Å^−3^
Δρ_min_ = −0.23 e Å^−3^

### 

Data collection: *SMART* (Bruker, 2007 ▶); cell refinement: *SAINT* (Bruker, 2007 ▶); data reduction: *SAINT*; program(s) used to solve structure: *SHELXS97* (Sheldrick, 2008 ▶); program(s) used to refine structure: *SHELXL97* (Sheldrick, 2008 ▶); molecular graphics: *SHELXTL* (Sheldrick, 2008 ▶); software used to prepare material for publication: *SHELXTL*.

## Supplementary Material

Crystal structure: contains datablocks global, I. DOI: 10.1107/S1600536810020039/zs2040sup1.cif

Structure factors: contains datablocks I. DOI: 10.1107/S1600536810020039/zs2040Isup2.hkl

Additional supplementary materials:  crystallographic information; 3D view; checkCIF report

## Figures and Tables

**Table 1:** Hydrogen-bond geometry (Å, °)

*D*—H⋯*A*	*D*—H	H⋯*A*	*D*⋯*A*	*D*—H⋯*A*
N2—H2*A*⋯O2^i^	0.86	2.03	2.876 (3)	170
C10—H10*B*⋯O3^ii^	0.97	2.45	3.311 (3)	148
C2—H2⋯O4^iii^	0.93	2.58	3.496 (3)	167

Symmetry codes: (i) 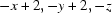; (ii) 
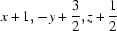
; (iii) 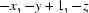.

**Fig. 1.**
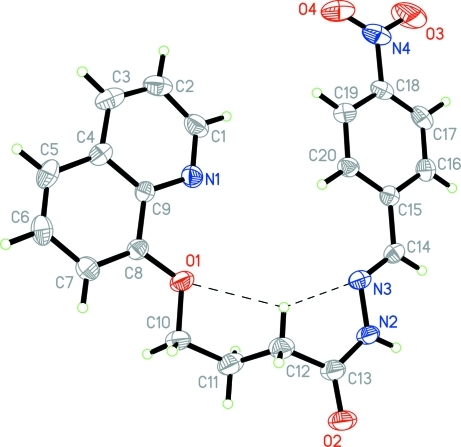
The molecular structure of (I), with displacement ellipsoids at the 30% probability level. Intramolecular C–H···N and C–H···O interactions are shown as dashed lines.

**Fig. 2.**
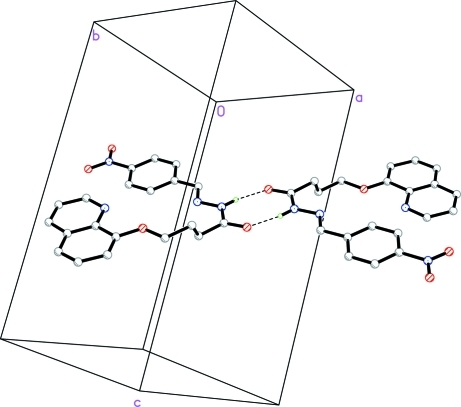
The cyclic hydrogen-bonded dimer with hydrogen bonds shown as dashed lines. H atoms, except for those involved in hydrogen bonds, are not included.

**Fig. 3.**
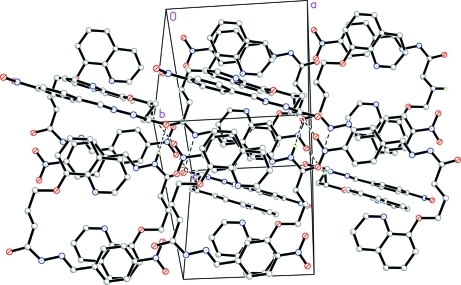
Part of the crystal structure showing hydrogen bonds as dashed lines. H atoms, except for those involved in hydrogen bonds, are not included.

## supplementary crystallographic information

### Comment

Schiff bases are one of the most prevalent mixed-donor ligands in the field of coordination chemistry, playing an important role in the development of the chemistry related to catalysis and enzymatic reactions, magnetism, and supramolecular architectures (Calligaris & Randaccio, 1987). Structures of Schiff bases derived from substituted 4-(quinolin-8-yloxy)butanehydrazide and closely related to the title compound have been reported earlier (Zheng, Li *et al.*, 2008; Zheng, Wu, Lu *et al.*, 2006; Zheng, 2006; Zheng, Wu, Li *et al.*, 2006, 2007; Xie *et al.*, 2008; Chen & Li, 2009; Zhang *et al.*, 2009). In this contribution, we present the synthesis and crystal structure of a new ligand C_20_H_18_N_4_O_4_ (I), which contains oxygen and nitrogen donors and a flexible aliphatic spacer.

In (I) (Fig.1) the asymmetric unit contains a crystallographically independent molecule with a *trans-*(+)*gauche-trans-trans-*(-)*gauche-trans-trans* conformation along the quinoline ring–benzene ring bond sequence [torsion angles (°): C8–O1–C10–C11, 179.83 (18); O1–C10–C11–C12, 65.1 (3); C10–C11–C12–C13, -178.24; C11–C12–C13–N2, 114.1 (2); C12–C13–N2–N3, -0.1 (3); C13–N2–N3–C14, -178.96 (7); N2–N3–C14–C15, -179.17 (16)]. The bond lengths and angles in (I) are in good agreement with the expected values (Allen *et al.*, 1987) and are comparable to those in the related compounds (Zheng, Wu, Lu *et al.*, 2006; Zheng, 2006; Zheng, Wu, Li *et al.*, 2006, 2007; Xie *et al.*, 2008; Chen *et al.*, 2009; Zhang, XiaHou *et al.*, 2009). The C14—N3 and C13—O2 bond lengths [1.269 (3) and 1.235 (2) Å, respectively] indicate the presence of a typical C═N and C═O. The C═N—N angle of 115.79 (18)° is significantly smaller than the ideal value of 120° expected for sp^2^-hybridized N atoms, probably due to repulsion between the nitrogen lone pairs and the adjacent N atom (Zheng, Qiu *et al.*, 2006). The benzene and quinoline ring systems are close to coplanar [dihedral angle, 2.7 (5)°]. In the crystal structure, intramolecular C—H···N and C—H···O interactions (Table 1, Fig. 1) produce two edge-sharing S(5) ring motifs (Bernstein *et al.*, 1995) and a pair of intermolecular N—H···O hydrogen bonds link the molecules into centrosymmetric cyclic R^2^_2_(8) dimers (Fig. 2), which are aggregated *via*π–π interactions into parallel sheets [quinoline–benzene ring centroid separation: 3.6173 (16)–3.6511 (16) Å], which are further connected through weak C—H···O interactions, giving a supramolecular two-dimensional network (Fig. 3).

### Experimental

Reagents and solvents used were of commercially available quality. The title compound (I) was synthesized according to the method of Zheng, Li *et al.*, 2008. 4-(Quinolin-8-yloxy)butanehydrazide (0.01 mol), *p*-formylnitrobenzene (0.01 mol), ethanol (40 ml) and some drops of acetic acid were added to a 100 ml flask and refluxed for 6 h. After cooling to room temperature, the solid product was separated by filtration. Yellow single crystals of (I) suitable for the X-ray diffraction study were obtained by slow evaporation of a tetrahydrofuran solution over a period of four days.

### Refinement

All H atoms were placed in idealized positions (C—H = 0.93–0.97 Å, N—H = 0.86 Å) and refined as riding atoms with *U*_iso_(H) = 1.2*U*_eq_(C or N).

### Crystal data

**Table:**

C_20_H_18_N_4_O_4_	*F*(000) = 792
*M**_r_* = 378.38	*D*_x_ = 1.369 Mg m^−^^3^
Monoclinic, *P*2_1_/*c*	Mo *K*α radiation, λ = 0.71073 Å
Hall symbol: -P 2ybc	Cell parameters from 3279 reflections
*a* = 9.836 (3) Å	θ = 2.2–27.9°
*b* = 10.633 (3) Å	µ = 0.10 mm^−^^1^
*c* = 17.566 (5) Å	*T* = 296 K
β = 92.365 (7)°	Prism, yellow
*V* = 1835.6 (9) Å^3^	0.22 × 0.17 × 0.15 mm
*Z* = 4	

### Data collection

**Table:**

Bruker SMART CCD area-detector diffractometer	3256 independent reflections
Radiation source: fine-focus sealed tube	2345 reflections with *I* > 2σ(*I*)
graphite	*R*_int_ = 0.036
φ and ω scans	θ_max_ = 25.0°, θ_min_ = 2.1°
Absorption correction: multi-scan (*SADABS*; Sheldrick, 1996)	*h* = −7→11
*T*_min_ = 0.979, *T*_max_ = 0.986	*k* = −12→12
9957 measured reflections	*l* = −20→20

### Refinement

**Table:**

Refinement on *F*^2^	Primary atom site location: structure-invariant direct methods
Least-squares matrix: full	Secondary atom site location: difference Fourier map
*R*[*F*^2^ > 2σ(*F*^2^)] = 0.044	Hydrogen site location: inferred from neighbouring sites
*wR*(*F*^2^) = 0.146	H-atom parameters constrained
*S* = 1.07	*w* = 1/[σ^2^(*F*_o_^2^) + (0.0719*P*)^2^ + 0.3873*P*] where *P* = (*F*_o_^2^ + 2*F*_c_^2^)/3
3256 reflections	(Δ/σ)_max_ < 0.001
253 parameters	Δρ_max_ = 0.22 e Å^−^^3^
0 restraints	Δρ_min_ = −0.22 e Å^−^^3^

### Special details

**Table:**

Geometry. All esds (except the esd in the dihedral angle between two l.s. planes) are estimated using the full covariance matrix. The cell esds are taken into account individually in the estimation of esds in distances, angles and torsion angles; correlations between esds in cell parameters are only used when they are defined by crystal symmetry. An approximate (isotropic) treatment of cell esds is used for estimating esds involving l.s. planes.
Refinement. Refinement of *F*^2^ against ALL reflections. The weighted *R*-factor wR and goodness of fit *S* are based on *F*^2^, conventional *R*-factors *R* are based on *F*, with *F* set to zero for negative *F*^2^. The threshold expression of *F*^2^ > σ(*F*^2^) is used only for calculating *R*-factors(gt) etc. and is not relevant to the choice of reflections for refinement. *R*-factors based on *F*^2^ are statistically about twice as large as those based on *F*, and *R*- factors based on ALL data will be even larger.

### Fractional atomic coordinates and isotropic or equivalent isotropic displacement parameters (Å^2^)

**Table:**

	*x*	*y*	*z*	*U*_iso_*/*U*_eq_	
O1	0.75513 (15)	0.53054 (15)	0.16112 (8)	0.0599 (4)	
O2	1.05475 (15)	0.89991 (18)	0.07985 (10)	0.0749 (5)	
O3	0.0388 (2)	0.8161 (3)	−0.16156 (15)	0.1302 (10)	
O4	0.04918 (18)	0.6940 (2)	−0.06516 (13)	0.0903 (6)	
N1	0.50202 (18)	0.48897 (16)	0.10959 (10)	0.0542 (5)	
N2	0.85327 (17)	0.90258 (17)	0.01739 (10)	0.0499 (4)	
H2A	0.8844	0.9544	−0.0152	0.060*	
N3	0.71999 (16)	0.86354 (16)	0.00912 (10)	0.0466 (4)	
N4	0.0993 (2)	0.7717 (2)	−0.10668 (13)	0.0710 (6)	
C1	0.3767 (2)	0.4663 (2)	0.08357 (15)	0.0651 (7)	
H1	0.3479	0.5042	0.0380	0.078*	
C2	0.2845 (2)	0.3894 (2)	0.11982 (17)	0.0685 (7)	
H2	0.1962	0.3793	0.0998	0.082*	
C3	0.3263 (2)	0.3297 (2)	0.18475 (15)	0.0642 (7)	
H3	0.2665	0.2777	0.2097	0.077*	
C4	0.4606 (2)	0.34635 (18)	0.21447 (11)	0.0472 (5)	
C5	0.5148 (3)	0.2828 (2)	0.28008 (12)	0.0601 (6)	
H5	0.4608	0.2271	0.3063	0.072*	
C6	0.6447 (3)	0.3036 (2)	0.30424 (12)	0.0605 (6)	
H6	0.6793	0.2610	0.3471	0.073*	
C7	0.7296 (2)	0.3877 (2)	0.26657 (11)	0.0521 (5)	
H7	0.8184	0.4012	0.2851	0.063*	
C8	0.6817 (2)	0.44914 (18)	0.20301 (11)	0.0444 (5)	
C9	0.5446 (2)	0.42934 (17)	0.17467 (10)	0.0410 (5)	
C10	0.8940 (2)	0.5533 (2)	0.18444 (13)	0.0587 (6)	
H10A	0.9454	0.4755	0.1842	0.070*	
H10B	0.8996	0.5879	0.2356	0.070*	
C11	0.9503 (2)	0.6450 (2)	0.12900 (15)	0.0663 (7)	
H11A	0.9384	0.6113	0.0779	0.080*	
H11B	1.0472	0.6550	0.1401	0.080*	
C12	0.8825 (2)	0.7721 (2)	0.13204 (13)	0.0615 (6)	
H12A	0.7853	0.7618	0.1225	0.074*	
H12B	0.8969	0.8070	0.1827	0.074*	
C13	0.9360 (2)	0.8621 (2)	0.07498 (13)	0.0561 (6)	
C14	0.6530 (2)	0.90707 (19)	−0.04826 (11)	0.0451 (5)	
H14	0.6949	0.9608	−0.0818	0.054*	
C15	0.5098 (2)	0.87299 (17)	−0.06186 (10)	0.0422 (5)	
C16	0.4388 (2)	0.9199 (2)	−0.12587 (12)	0.0528 (5)	
H16	0.4825	0.9730	−0.1591	0.063*	
C17	0.3046 (2)	0.8885 (2)	−0.14041 (13)	0.0581 (6)	
H17	0.2572	0.9203	−0.1831	0.070*	
C18	0.2416 (2)	0.8095 (2)	−0.09103 (12)	0.0503 (5)	
C19	0.3084 (2)	0.7626 (2)	−0.02718 (12)	0.0518 (5)	
H19	0.2635	0.7104	0.0060	0.062*	
C20	0.4429 (2)	0.79389 (19)	−0.01275 (11)	0.0475 (5)	
H20	0.4892	0.7618	0.0302	0.057*	

### Atomic displacement parameters (Å^2^)

**Table:**

	*U*^11^	*U*^22^	*U*^33^	*U*^12^	*U*^13^	*U*^23^
O1	0.0511 (9)	0.0703 (10)	0.0573 (9)	−0.0236 (8)	−0.0086 (7)	0.0125 (8)
O2	0.0459 (9)	0.1000 (13)	0.0779 (11)	−0.0223 (9)	−0.0080 (8)	0.0215 (10)
O3	0.0686 (13)	0.181 (2)	0.1362 (19)	0.0046 (16)	−0.0528 (14)	0.0321 (19)
O4	0.0516 (10)	0.1168 (17)	0.1021 (15)	−0.0198 (11)	−0.0025 (10)	−0.0187 (13)
N1	0.0546 (11)	0.0479 (10)	0.0592 (11)	−0.0031 (9)	−0.0103 (9)	0.0030 (8)
N2	0.0389 (9)	0.0549 (10)	0.0559 (10)	−0.0102 (8)	0.0021 (8)	0.0007 (8)
N3	0.0387 (9)	0.0472 (10)	0.0541 (10)	−0.0061 (8)	0.0047 (8)	−0.0070 (8)
N4	0.0460 (11)	0.0887 (16)	0.0770 (14)	0.0091 (12)	−0.0111 (11)	−0.0183 (13)
C1	0.0564 (14)	0.0588 (14)	0.0781 (16)	0.0060 (12)	−0.0205 (12)	−0.0039 (12)
C2	0.0432 (12)	0.0687 (16)	0.0928 (19)	0.0000 (12)	−0.0065 (13)	−0.0211 (14)
C3	0.0516 (13)	0.0623 (14)	0.0801 (17)	−0.0155 (12)	0.0208 (12)	−0.0221 (13)
C4	0.0523 (12)	0.0399 (11)	0.0505 (11)	−0.0051 (10)	0.0143 (9)	−0.0121 (9)
C5	0.0829 (17)	0.0493 (13)	0.0499 (12)	−0.0090 (12)	0.0230 (12)	−0.0003 (10)
C6	0.0850 (18)	0.0582 (13)	0.0385 (11)	0.0083 (13)	0.0041 (11)	0.0062 (10)
C7	0.0559 (12)	0.0575 (13)	0.0425 (11)	0.0029 (11)	−0.0035 (9)	−0.0026 (10)
C8	0.0461 (11)	0.0435 (10)	0.0435 (11)	−0.0048 (9)	0.0020 (9)	−0.0023 (9)
C9	0.0455 (11)	0.0357 (10)	0.0416 (10)	−0.0021 (9)	0.0014 (8)	−0.0041 (8)
C10	0.0422 (12)	0.0608 (13)	0.0725 (15)	−0.0079 (11)	−0.0037 (11)	−0.0013 (11)
C11	0.0520 (13)	0.0645 (15)	0.0831 (16)	−0.0131 (12)	0.0126 (12)	−0.0032 (13)
C12	0.0461 (12)	0.0765 (16)	0.0622 (14)	−0.0066 (12)	0.0054 (10)	0.0092 (12)
C13	0.0434 (12)	0.0658 (14)	0.0590 (13)	−0.0086 (11)	0.0026 (10)	0.0016 (11)
C14	0.0462 (11)	0.0437 (11)	0.0458 (11)	−0.0065 (9)	0.0059 (9)	−0.0052 (9)
C15	0.0454 (11)	0.0391 (10)	0.0422 (10)	0.0025 (9)	0.0026 (9)	−0.0075 (8)
C16	0.0618 (13)	0.0492 (12)	0.0473 (11)	0.0026 (11)	0.0006 (10)	0.0035 (9)
C17	0.0603 (14)	0.0613 (14)	0.0514 (12)	0.0178 (12)	−0.0125 (11)	−0.0031 (11)
C18	0.0391 (10)	0.0548 (12)	0.0563 (12)	0.0081 (10)	−0.0063 (9)	−0.0134 (10)
C19	0.0436 (11)	0.0564 (13)	0.0555 (12)	−0.0039 (10)	0.0018 (9)	−0.0008 (10)
C20	0.0430 (11)	0.0543 (12)	0.0448 (11)	−0.0013 (10)	−0.0047 (9)	0.0012 (9)

### Geometric parameters (Å, °)

**Table:**

O1—C8	1.363 (2)	C7—C8	1.361 (3)
O1—C10	1.430 (2)	C7—H7	0.9300
O2—C13	1.235 (2)	C8—C9	1.433 (3)
O3—N4	1.208 (3)	C10—C11	1.500 (3)
O4—N4	1.220 (3)	C10—H10A	0.9700
N1—C1	1.319 (3)	C10—H10B	0.9700
N1—C9	1.358 (2)	C11—C12	1.508 (3)
N2—C13	1.343 (3)	C11—H11A	0.9700
N2—N3	1.377 (2)	C11—H11B	0.9700
N2—H2A	0.8600	C12—C13	1.497 (3)
N3—C14	1.269 (3)	C12—H12A	0.9700
N4—C18	1.471 (3)	C12—H12B	0.9700
C1—C2	1.394 (4)	C14—C15	1.464 (3)
C1—H1	0.9300	C14—H14	0.9300
C2—C3	1.354 (4)	C15—C20	1.390 (3)
C2—H2	0.9300	C15—C16	1.392 (3)
C3—C4	1.412 (3)	C16—C17	1.375 (3)
C3—H3	0.9300	C16—H16	0.9300
C4—C9	1.413 (3)	C17—C18	1.374 (3)
C4—C5	1.421 (3)	C17—H17	0.9300
C5—C6	1.348 (3)	C18—C19	1.370 (3)
C5—H5	0.9300	C19—C20	1.377 (3)
C6—C7	1.407 (3)	C19—H19	0.9300
C6—H6	0.9300	C20—H20	0.9300
			
C8—O1—C10	118.32 (16)	O1—C10—H10B	110.2
C1—N1—C9	117.24 (19)	C11—C10—H10B	110.2
C13—N2—N3	121.83 (18)	H10A—C10—H10B	108.5
C13—N2—H2A	119.1	C10—C11—C12	112.5 (2)
N3—N2—H2A	119.1	C10—C11—H11A	109.1
C14—N3—N2	115.79 (18)	C12—C11—H11A	109.1
O3—N4—O4	123.0 (2)	C10—C11—H11B	109.1
O3—N4—C18	118.4 (3)	C12—C11—H11B	109.1
O4—N4—C18	118.6 (2)	H11A—C11—H11B	107.8
N1—C1—C2	124.4 (2)	C13—C12—C11	112.35 (19)
N1—C1—H1	117.8	C13—C12—H12A	109.1
C2—C1—H1	117.8	C11—C12—H12A	109.1
C3—C2—C1	118.6 (2)	C13—C12—H12B	109.1
C3—C2—H2	120.7	C11—C12—H12B	109.1
C1—C2—H2	120.7	H12A—C12—H12B	107.9
C2—C3—C4	120.0 (2)	O2—C13—N2	119.3 (2)
C2—C3—H3	120.0	O2—C13—C12	121.3 (2)
C4—C3—H3	120.0	N2—C13—C12	119.38 (19)
C3—C4—C9	116.9 (2)	N3—C14—C15	120.28 (19)
C3—C4—C5	123.6 (2)	N3—C14—H14	119.9
C9—C4—C5	119.41 (19)	C15—C14—H14	119.9
C6—C5—C4	119.8 (2)	C20—C15—C16	118.90 (19)
C6—C5—H5	120.1	C20—C15—C14	121.72 (18)
C4—C5—H5	120.1	C16—C15—C14	119.38 (19)
C5—C6—C7	121.9 (2)	C17—C16—C15	120.6 (2)
C5—C6—H6	119.0	C17—C16—H16	119.7
C7—C6—H6	119.0	C15—C16—H16	119.7
C8—C7—C6	119.9 (2)	C18—C17—C16	119.1 (2)
C8—C7—H7	120.0	C18—C17—H17	120.5
C6—C7—H7	120.0	C16—C17—H17	120.5
C7—C8—O1	125.06 (19)	C19—C18—C17	121.7 (2)
C7—C8—C9	120.23 (19)	C19—C18—N4	118.3 (2)
O1—C8—C9	114.71 (16)	C17—C18—N4	120.0 (2)
N1—C9—C4	122.77 (18)	C18—C19—C20	119.2 (2)
N1—C9—C8	118.49 (17)	C18—C19—H19	120.4
C4—C9—C8	118.73 (17)	C20—C19—H19	120.4
O1—C10—C11	107.34 (19)	C19—C20—C15	120.53 (19)
O1—C10—H10A	110.2	C19—C20—H20	119.7
C11—C10—H10A	110.2	C15—C20—H20	119.7
			
C13—N2—N3—C14	−178.96 (19)	C8—O1—C10—C11	179.83 (18)
C9—N1—C1—C2	2.4 (3)	O1—C10—C11—C12	65.1 (3)
N1—C1—C2—C3	−2.5 (4)	C10—C11—C12—C13	−178.24 (19)
C1—C2—C3—C4	0.2 (3)	N3—N2—C13—O2	180.00 (19)
C2—C3—C4—C9	1.7 (3)	N3—N2—C13—C12	−0.1 (3)
C2—C3—C4—C5	−176.9 (2)	C11—C12—C13—O2	−66.0 (3)
C3—C4—C5—C6	179.5 (2)	C11—C12—C13—N2	114.1 (2)
C9—C4—C5—C6	0.9 (3)	N2—N3—C14—C15	−179.17 (16)
C4—C5—C6—C7	0.4 (3)	N3—C14—C15—C20	0.6 (3)
C5—C6—C7—C8	−1.1 (3)	N3—C14—C15—C16	−178.84 (18)
C6—C7—C8—O1	−178.65 (19)	C20—C15—C16—C17	−0.1 (3)
C6—C7—C8—C9	0.5 (3)	C14—C15—C16—C17	179.41 (18)
C10—O1—C8—C7	0.7 (3)	C15—C16—C17—C18	−0.3 (3)
C10—O1—C8—C9	−178.53 (17)	C16—C17—C18—C19	0.9 (3)
C1—N1—C9—C4	−0.3 (3)	C16—C17—C18—N4	−178.20 (19)
C1—N1—C9—C8	178.11 (19)	O3—N4—C18—C19	177.6 (2)
C3—C4—C9—N1	−1.7 (3)	O4—N4—C18—C19	−4.7 (3)
C5—C4—C9—N1	176.92 (18)	O3—N4—C18—C17	−3.2 (3)
C3—C4—C9—C8	179.88 (18)	O4—N4—C18—C17	174.4 (2)
C5—C4—C9—C8	−1.5 (3)	C17—C18—C19—C20	−1.1 (3)
C7—C8—C9—N1	−177.70 (18)	N4—C18—C19—C20	178.07 (18)
O1—C8—C9—N1	1.6 (3)	C18—C19—C20—C15	0.6 (3)
C7—C8—C9—C4	0.8 (3)	C16—C15—C20—C19	−0.1 (3)
O1—C8—C9—C4	−179.99 (16)	C14—C15—C20—C19	−179.55 (18)

### Hydrogen-bond geometry (Å, °)

**Table:**

*D*—H···*A*	*D*—H	H···*A*	*D*···*A*	*D*—H···*A*
N2—H2A···O2^i^	0.86	2.03	2.876 (3)	170
C12—H12A···O1	0.97	2.57	2.912 (3)	101
C12—H12A···N3	0.97	2.33	2.807 (3)	109
C10—H10B···O3^ii^	0.97	2.45	3.311 (3)	148
C2—H2···O4^iii^	0.93	2.58	3.496 (3)	167

Symmetry codes: (i) −*x*+2, −*y*+2, −*z*; (ii) *x*+1, −*y*+3/2, *z*+1/2; (iii) −*x*, −*y*+1, −*z*.
